# Chemical and Thermo-Mechanical Properties of Waterborne Polyurethane Dispersion Derived from Jatropha Oil

**DOI:** 10.3390/polym13050795

**Published:** 2021-03-05

**Authors:** Sariah Saalah, Luqman Chuah Abdullah, Min Min Aung, Mek Zah Salleh, Dayang Radiah Awang Biak, Mahiran Basri, Emiliana Rose Jusoh, Suhaini Mamat, Syeed SaifulAzry Osman Al Edrus

**Affiliations:** 1 Chemical Engineering Programme, Faculty of Engineering, Universiti Malaysia Sabah, Jalan UMS, Kota Kinabalu 88400, Sabah, Malaysia; 2 Department of Chemical and Environmental Engineering, Faculty of Engineering, Universiti Putra Malaysia, Serdang 43400, Selangor, Malaysia; dradiah@upm.edu.my; 3 Higher Institution Centre of Excellence Wood and Tropical Fibre (HICoE), Institute of Tropical Forestry and Forest Products, Universiti Putra Malaysia, Serdang 43400, Selangor, Malaysia; minmin_aung@upm.edu.my (M.M.A.); tintahikmah@gmail.com (E.R.J.); saifulazry@upm.edu.my (S.S.O.A.E.); 4 Department of Chemistry, Faculty of Science and Technology, Universiti Putra Malaysia, Serdang 43400, Selangor, Malaysia; mahiran@science.upm.edu.my; 5 Radiation Processing Technology Division, Malaysian Nuclear Agency, Kajang 43000, Selangor, Malaysia; mekzah@nuclearmalaysia.gov.my; 6 School of Engineering Technology, Malaysian Institute of Chemical and Bioengineering Technology (UniKL MICET), Universiti Kuala Lumpur, Taboh Naning, Alor Gajah 78000, Melaka, Malaysia; suhainim@micet.unikl.edu.my

**Keywords:** waterborne polyurethane dispersion, water-based coatings, jatropha oil, crosslinking density

## Abstract

Nowadays, there is a significant trend away from solvent-based polyurethane systems towards waterborne polyurethane dispersions due to government regulations requiring manufacturers to lower total volatile organic compounds, as well as consumer preference for more environmentally friendly products. In this work, a renewable vegetable oil-based polyol derived from jatropha oil was polymerized with isophorone diisocyanate and dimethylol propionic acid to produce anionic waterborne polyurethane dispersion. Free standing films with up to 62 wt.% bio-based content were successfully produced after evaporation of water from the jatropha oil-based waterborne polyurethane (JPU) dispersion, which indicated good film formation. The chemical and thermo-mechanical properties of the JPU films were characterized. By increasing the OH numbers of polyol from 161 mgKOH/g to 217 mgKOH/g, the crosslinking density of the JPU was significantly increased, which lead to a better storage modulus and improved hydrophobicity. Overall, JPU produced from polyol having OH number of 217 mgKOH/g appears to be a promising product for application as a binder for wood and decorative coatings.

## 1. Introduction

Waterborne polyurethane (WBPU) is rapid growing segment of the polyurethane (PU) industry due to the increasing worldwide concern about the environmental conditions caused by the volatile organic compound (VOC) content in traditional polyurethane coatings. In coating application, a solvent is essential to carry the coating from applicator to the substrate, which then evaporates to produce a dry coating film. The organic solvent evaporation contributes significantly to the VOC emissions in the atmosphere. Conventional coatings and adhesives are typically formulated based on 40–60% wt. volatile organic solvents, which can cause environmental problems and is harmful to the body, causing irritation to the eyes, nose and throat as well as dizziness and headache [1]. The coatings industry is the largest user of organic solvents, while one-third of the total raw material cost is that of solvents [2].

Replacing traditional solvent with water in PU coating formulations is a more environmentally friendly approach which benefits the workers during application as it is easy to handle, non-toxic and reduces the risk of fire during storage and transportation. WBPU contains hydrophilic ionic centres at its backbone to allow dispersion of hydrophobic PU in water, thus forming a stable colloid. These products fulfil many of the requirements related to conventional solvent-borne coatings, e.g., low viscosity at a high molecular weight and good applicability [3]. WBPU shows high adhesion with various substrates such as textiles, paper, leather, polymers and glass. WBPU creates a clear and transparent plastic coat, emits fewer odours and dries very fast. Commercial solvent-based polyurethane creates a yellowish or slightly amber-coloured plastic coating on the wooden surface and requires more time to dry [4]. Coating segments accounted for 47% of the total global consumption of polyurethane dispersion in 2016, and the share is expected to grow at a CAGR of 6.98% by 2023 [5].

On the other hand, there is growing interest nowadays in producing WBPU from bio-based raw materials. In producing polyurethane, polyol and diisocyanate are the main ingredients. Vegetable oils have emerged as a green alternative source for the polyols or the precursors to the synthesis of this component, due to their availability, low eco-toxicity, relatively low cost and biodegradability [6]. Several vegetable oils have been used to synthesize bio-based polyols, such as cottonseed oil, soybean oil, castor oil, sunflower oil, canola oil, corn oil, palm oil, rapeseed oil, jatropha oil and tung oil, among others [7,8,9]. Accordingly, most of these bio-polyols have been successfully used to synthesize WBPU [9,10,11,12,13]. However, limited works have been reported on waterborne polyurethane dispersion synthesized from jatropha oil.

Jatropha oil, which is extracted from the seeds of the jatropha fruit, is a promising candidate for chemical purposes as it contains 78.9% unsaturated fatty acids, mainly of oleic acid (43.1%) and linoleic acid (34.4%) [14]. This high degree of unsaturation provides a broad alternative for chemical modification to produce polymers with the desired properties. Furthermore, the use of a non-edible jatropha oil will reduce the dependency on edible oils for chemical purposes [15]. Previous research has revealed the potential usage of jatropha oil for production of various polymers with promising properties such as alkyd resin, PU coatings, PU adhesive and PU elastomer [16,17,18,19]. 

In our previous works, WBPU synthesized from jatropha oil-based polyols were reported, which focused on the wet characteristics such as particle size, zeta potential and rheology. The results show excellent colloidal stability of the dispersion and the rheology is comparable to petrochemical-based WBPU [20]. The colloidal stability and rheology of the dispersion are important to determining storage stability and applicability of the coatings on substrate [21]. However, the characteristics of the colloidal dispersions do not directly affect the mechanical properties of the resulting dry films. It was reported that the ionic groups of the emulsifier tend to improve the mechanical properties but the films become more sensitive to water and chemicals [22]. On the other hand, formulation of PU with a balanced composition between soft segment (polyol) and the hard segment will determine the mechanical properties of the polymers [23]. The hardness and tensile properties of the resulting WBPU films from jatropha oil are comparable to commercial petro-based WBPU dispersion wood coatings [24,25]. Therefore, the current works aimed to determine the chemical and thermo-mechanical performance of the jatropha oil-based WBPU films. These properties will have an influence on the practical design of products as the PU dispersion can be used as a standalone coating or as a binder in wood and decorative coatings. 

## 2. Materials and Method

### 2.1. Materials

Reagent grade hydrogen peroxide 30% and methanol were supplied by Merck, Germany. Meanwhile, isophorone diisocyanate (IPDI) 98%, dimethylol propionic acid (DMPA), n-methyl pyrollidone (NMP), hydroxyethyl metachrylate (HEMA), phtalic anhydride and dibutyltin dilaurate (DBTDL) were supplied by Sigma Aldrich (Milwaukee, WI, USA). Ethyl Methyl Ketone (MEK), triethylamine (TEA), formic acid, magnesium sulphate anhydrous, pyridine and sodium hydroxide were reagent grades, supplied by Classic Chemicals (Shah Alam, Malaysia). Besides that, the crude jatropha oil was supplied by Bionas Sdn Bhd, Kuala Lumpur, Malaysia, which is a non-food grade material, and was used as received. The Jatropha oil-based polyol were synthesized by epoxidation followed by ring opening step, and the detailed procedure has been described in our previous work [26].

### 2.2. Synthesis of Jatropha Oil-Based Waterborne Polyurethane (JPU) Dispersions 

**Figure 1:**
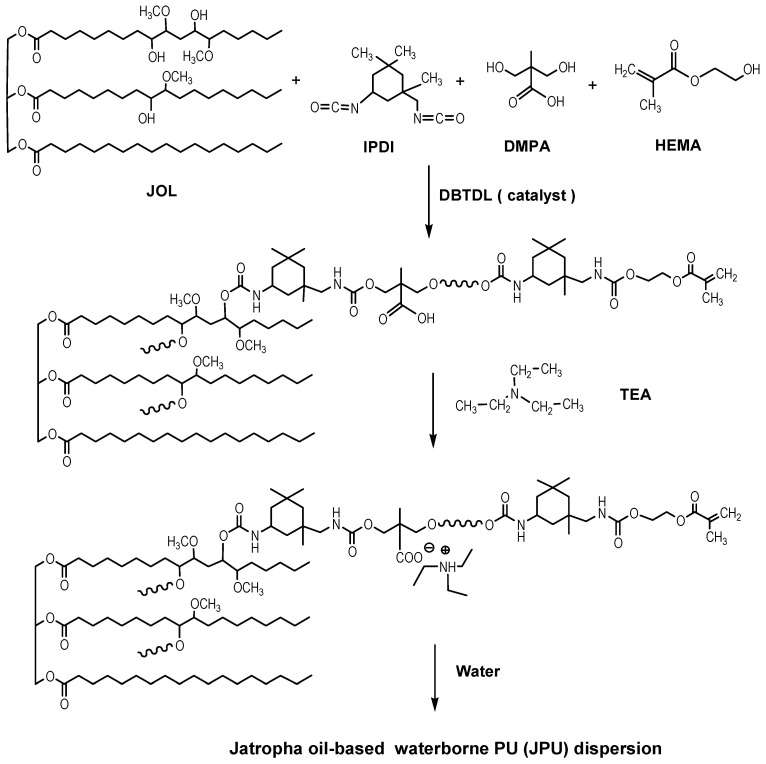
Schematic for the synthesis of Jatropha oil -based waterborne polyurethane (JPU) dispersions.

Jatropha oil-based waterborne PU (JPU) dispersions were synthesised by the acetone process according to the method described in our previous works [20,25]. The schematic for the synthesis of jatropha oil-based waterborne polyurethane (JPU) dispersions is shown in Figure 1. Briefly, jatropha oil-based polyol (JOL) and DMPA (dissolved in NMP) were added to a four-necked flask equipped with a mechanical stirrer, dropping funnel, condenser and thermometer. 

The mixture was heated to 78 °C and stirred for 30 min to obtain a homogeneous mixture. IPDI was then added dropwise for 30 min, followed by a few drops of dibutyltn dilaurate as a catalyst. MEK was added batch by batch to reduce the viscosity of the system. After an additional 3 h of reaction, HEMA was added as a chain terminating agent. The reaction was carefully monitored by FTIR analysis. The disappearance of an NCO peak at 2270 cm^−1^ of ATR-FTIR spectra was used as indicator that all diisocyanate was consumed in the reaction. The reactants were then cooled to 40 °C and neutralized by the adding TEA (1.2 equiv. per DMPA), followed by dispersion with distilled water at high speed (1000–1800 rpm). The JPU dispersions with a solid content of ~25 wt.% was produced after removal of the MEK under vacuum.

**Figure 2:**
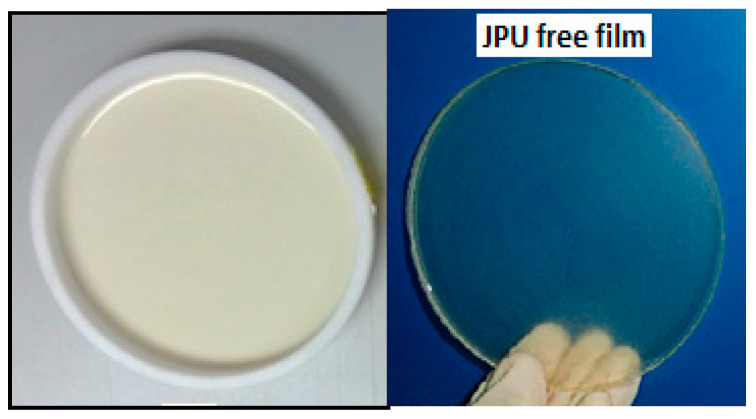
JPU film produced by casting the dispersion in Teflon mold.

The PU films were obtained by casting the PU dispersions into a teflon mold, and drying them at room temperature for 7 days, followed by drying in vacuum oven at 60 °C for 12 h [3]. The JPU films were stored in a desiccator at room temperature for further analysis and characterization. The thickness of the resulting polyurethane films was about 0.6–1.0 mm. It is worth mentioning that PU dispersions had to be cast on a Teflon surface due to high adhesiveness of the films observed on the glass, aluminium and plastic surface, making demoulding impossible. Figure 2 shows JPU dispersions cast in the Teflon mould for the preparation of film. The resulting films are clear and transparent, which is the common appearance of WBPU and other film coatings [27,28].

Table 1 shows the formulations of waterborne JPU. The “JPU 217” designation indicated that the hydroxyl number of polyol used for JPU preparation is 217 mgKOH/g. The molar ratio of the polyol, DMPA, IPDI and HEMA in all formulations were fixed which leads to PUs with an increase in DMPA content (wt.%) and hard segment content (wt.%).

### 2.3. Characterisation of Jatropha Oil-Based Waterborne (JPU) Films

#### 2.3.1. Fourier Transform Infrared Spectroscopy (FTIR) Analysis

The ATR-FTIR spectra of the films were recorded on a Perkin-Elmer Spectrum 2000 spectrometer (Perkin Elmer, Norwalk, CT, USA). A small sample was cut from the film and placed on the ATR accessories. The spectra were recorded in a range of 4000–500 cm^−1^.

#### 2.3.2. Crosslinking Density Measurement

Crosslinking density of the JPU films was performed according to method described by Zlatanic et al. [29]. A known weight (*W*_0_) of the films were immersed in a toluene at 23 °C for 7 days. Specimens were taken out and both surfaces were dried with a towel prior to weight measurement. The towel-dried sample weight ($W_{1}$) and the oven-dried film weight ($W_{2}$) were obtained. Three measurements were averaged for each sample. The percentage of the swell and the weight losses of the JPU films in toluene were calculated according to Equations (1) and (2), respectively. The insoluble part gives the gel fraction (Equation (3)), with $W_{g}$ is the weight of the gel.

(1) $$Swell\;\left(\%\right)=\frac{W_{1}-W_{0}}{W_{0}}\times 100\;$$

(2) $$Weight\;loss\;in\;toluene\;\left(\%\right)=\frac{W_{0}-W_{2}}{W_{0}}\times 100\;$$

(3) $$Gel\;\left(\%\right)=\left(\frac{W_{g}}{W_{o}}\right)\times 100\%\;$$

On the other hand, crosslinking density, υ_e_ as well as the molecular weight of polymer between cross-links, *M_c_*, can be determined according to the well-known Flory-Rehner theory based on the affine network [30] as in the Equation (4).

(4) $$\frac{1}{\vartheta_{e}}=\frac{M_{c}}{\rho_{2}}=\frac{-v_{1}\left(\emptyset_{2}{}^{1/3}-\frac{2\emptyset_{2}}{f}\right)}{\ln\left(1-\emptyset_{2}\right)+\emptyset_{2}+\chi_{12}\emptyset_{2}}\;$$

Here, $\rho_{2}$ is density of dry polymer, *v*_1_ is molar volume of the solvent (106.3 mL/mol for toluene) and *f* is the functionality of the network branch points. The volume fraction of polymer in the swollen network, $\emptyset_{2}$, is calculated according to Equation (5) [30], while the polymer–solvent interaction parameter, $\chi_{12}$ is estimated from the solubility parameters for the solvent and the polymer network (Equation (6)).

(5) $$\emptyset_{2}=\frac{\frac{W_{g}}{\rho_{2}}}{\frac{W_{g}}{\rho_{2}}+\frac{W_{s}}{\rho_{s}}}\;$$

(6) $$\chi_{12}=\frac{{\left(\delta_{1}-\delta_{2}\right)}^{2}v_{1}}{RT}\;$$

where *W_g_* is the weight of the gel, *W_s_* is the weight of the solvent in gel, *ρ* is the density of the solvent and *δ*_1_ is a solubility parameter of solvent (18.3 J^1/2^/cm^3/2^ for toluene). Solubility parameter of the polymer network, *δ*_2_, is calculated from the Hoy values, for the molar concentration constant, F, of the groups present in the polymer [31].

#### 2.3.3. Water Contact Angle Measurement

The contact angle between a water drop and the surface of the sample was measured using a contact-angle meter (FACE, Kyowa Interface Science Co. Ltd., Niiza, Japan). The drop of water was mounted on the surface of the films using a micro syringe and the contact angle was measured. The measurements were done in triplicate on different parts of the films.

#### 2.3.4. Water Uptake Determination

The water uptake of the JPU films were determined according to a method described by Fang et al. [32], with a slight modification. In this measurement, the films were immersed in deionized water for 5 days at room temperature. The weight of the swollen samples was recorded every 1 h for the first 10 h, followed by larger time intervals as the swelling proceeds to equilibrium. After each time interval, the sample was taken out and the surface was wiped using tissue paper to remove water, followed by immediate weighing of the swollen samples. The percentage of water uptake for a particular film was determined by measuring its weight changes, according to Equation (7):

(7) $$Water\;uptake\;\left(\%\right)=\frac{W-W_{0}}{W_{0}}\times 100\;$$

where $W_{0}$ is the weight of the dried film and W is the weight after water absorption. The measurements were done in triplicate.

#### 2.3.5. Differential Scanning Calorimetry (DSC) Analysis

Differential Scanning Calorimetry (DSC) was performed on DSC 823e (Mettler-Toledo, Greifensee, Switzerland) according to ASTM D3418-03. The measurement was performed according to the method described by Lu and Larock, [33]. The films (5–10 mg) were heated from 25 to 100 °C at a rate of 10 °C/min to erase previous thermal history, cooled to −70 at a rate of 10 °C/min, and heated again to 150 °C at a rate of 10 °C/min under nitrogen atmosphere. The heat flow curves were analysed on STARe software version 9.10 Mettler. The glass transition temperature (T_g_) of the film samples was determined from the midpoint temperature in the heat capacity change of the second DSC scan.

#### 2.3.6. Dynamic Mechanical Analysis (DMA)

Dynamic mechanical analysis (DMA) was carried out on DMA Q800 V20.24 (TA InstrumentsNew Castle, DE, USA) according to ASTM D5062-01 standard practice (ASTM Standard D5062-01 2001). A rectangular specimen of 10 mm × 5 mm × 0.5 mm (length × width × height) was analysed under a tension mode configuration at 1 Hz, a heating rate of 5 °C/min, at the temperature range of −60 to 100 °C. The storage modulus (E’), loss modulus (E”) and loss factor (tan δ) of the JPU films were measured as a function of temperature.

## 3. Results and Discussion

### 3.1. Drying Characteristic

**Figure 3:**
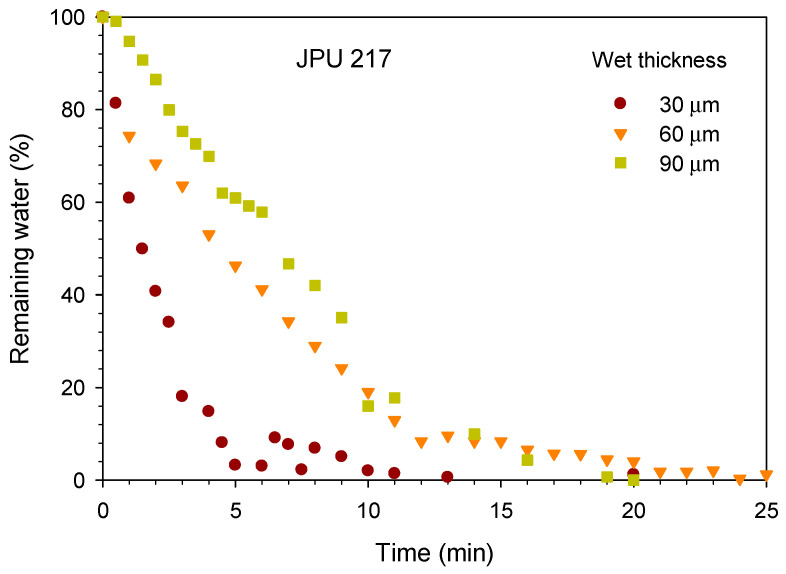
Effect of wet thickness on the drying time of JPU 217.

**Figure 4:**
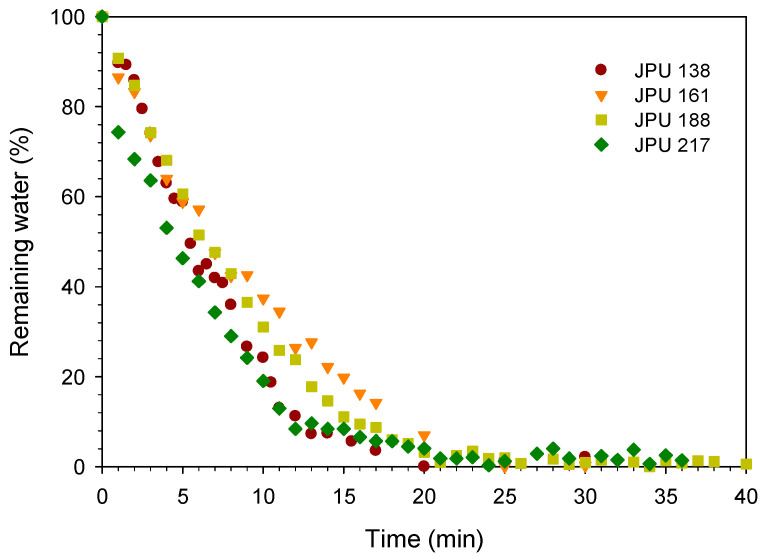
Effect of JPU variations on drying time.

The jatropha oil-based waterborne polyurethane (JPU) dispersions were dried and formed a film by evaporation of water and consequently the particles coalesced. In the water flash-off step, the physical entanglements occurred during the aqueous dispersion to become a tack-free dried film. The drying time of the JPU dispersions was measured by gravimetry upon water evaporation as shown in Figure 3. The water drying rate increased with reducing wet thickness. The water dried quickly from the beginning of the experiment and residual water was nearly zero after 20 min at 25 °C. For JPU dispersions having four different OH number polyols (Figure 4), no significant trend was observed, and all the samples were dried after 25 min at 25 °C.

The film formation mechanism has been reported [34,35]. Typical form formation behaviour during drying of latex includes three stages;

Stage 1: Once applied to a substrate, the water and solvents within the emulsion begin to evaporate, leading to a close-packed layer of latex particles. The rate of evaporation is approximately equal to the rate of evaporation of water.

Stage 2: The second stage begins when the particles are concentrated and come into irreversible contact. A clear, continuous, but still weak film is formed due to particle deformation at temperatures greater than the minimum film forming temperature (MFFT). The MFFT is the lowest temperature at which coalescence occurs sufficiently to form a continuous film.

Stage 3: The final stage of film formation occurs at temperatures above the glass transition temperature (T_g_). Further coalescence transpires as polymer surface chains interdiffuse across interfaces of adjacent particles to form a homogeneous polymer film, where this process is also called aging [34]. In addition, interdiffusion develops the mechanically coherent film. A long time is needed for the slow diffusion process of the polymer molecules across the polymer–polymer interfaces, time scales of days or even months up to one year have been reported [34,35].

In this work, the drying time was reported based on the first stage of film formation only. After evaporation of water from the dispersion, it was observed that the films which contained 55 to 62 wt.% polyol (JOL) as the renewable bio-based component (or equivalent to 45 to 38 wt.% hard segment) tended to dry to a glossy and transparent free standing film. Unfortunately, JPU 138 with 66 wt.% JOL content remained sticky and soft, indicating poor structural rigidity. The results suggested that sufficient OH functionality is required to obtain good film performance by increasing the urethane bond between the soft to hard segment components. In this case, the maximum allowable JOL content in PU was 62 wt.% with a minimum OH number of 161 mg KOH/g for production of a free-standing film. Therefore, JPU 138 film was not tested for mechanical properties.

### 3.2. Structure Analysis by FTIR

FTIR has been used to investigate the structure of the jatropha oil-based waterborne polyurethane (JPU) films. The JPUs were composed of a soft segment of jatropha oil-based polyol and a hard segment which consisted of urethane linkages generated by the reaction of diisocyanate, an internal emulsifier and a chain terminating agent. The properties of the PU were very much dependent on the hydrogen bonding interaction between the hard-to-hard segment and the hard-to soft segment through hydrogen bonding. The strength of the hydrogen bonding in the hard-to-hard segment was stronger than the bond in the hard-to-soft segment [3]. In solid film, hydrogen bonding causes the aggregation of these hard segments to form a hard domain [36]. JPU are capable of forming hydrogen bonds due to the presence of the amide hydrogen (N-H) group as a proton donor, and urethane carbonyl (C=O), ether oxygen (-O-) as well as the carbonyl group of the JOL as an acceptor in the urethane linkage.

**Figure 5:**
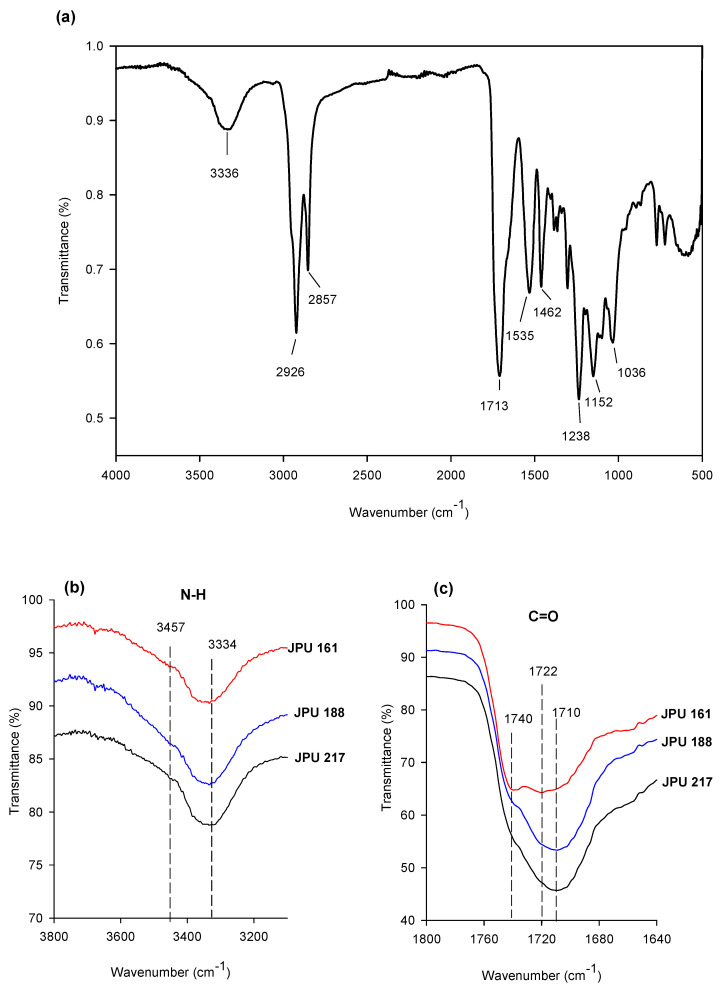
FTIR spectra of (**a**) JPU 217 film, (**b**) amide region of JPU films, and (**c**) carbonyl regions of JPU films.

Figure 5a shows the FTIR spectra of the JPU 217 film, and the peak assignment is tabulated in Table 2 [37]. The absorption bands observed at 3336 cm^−1^ and 1710 cm^−1^ corresponded to N-H (hydrogen bonded) and C=O (hydrogen bonded) respectively, which are the main characteristics of polyurethane. The spectra of the N-H stretching as well as the C=O stretching vibration region of the JPU from different OH polyol is presented in Figure 5b,c. A small shoulder peak was observed at 3457 cm^−1^ which was attributed to non-hydrogen-bonded N-H stretching, is relatively weak, implying that most of the amide groups in the JPU films were involved in hydrogen bonding [33].

The FTIR spectra of the C=O stretching region appeared to be composed of three bands at around 1740, 1723 and 1710 cm^−1^. Absorbance at 1740 cm^−1^ corresponded to free carbonyl stretching, while absorbance at 1723 and 1710 cm^−1^ was attributed to hydrogen bonding of carbonyl stretching. The free carbonyl group was only prominent in JPU 161, while the intensity of the hydrogen bonded carbonyl increased with OH number from 161 to 217 mg KOH/g which suggested an increasing intermolecular hydrogen bonding interaction between the soft and hard segments. The higher OH functionality of the polyol as the soft segment led to the formation of more urethane linkages and crosslinking in the resulting polymer. On the other hand, it is worth mentioning that the crystalline region due to the presence of ordered hydrogen bonding was not observed in the spectra, suggesting the amorphous nature of JPUs [33].

### 3.3. Crosslinking Density

**Figure 6:**
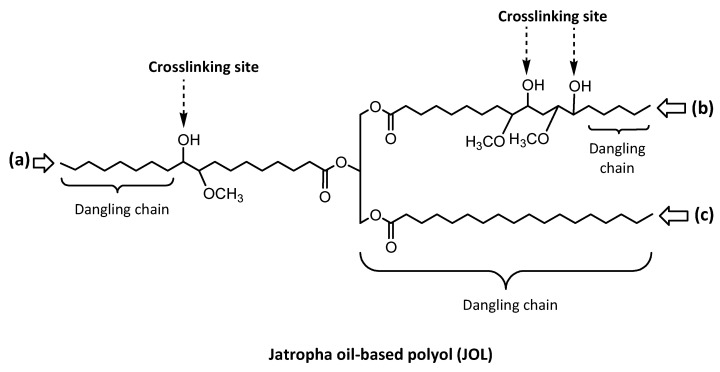
Position of reactive site at the fatty acid chain of polyol: (**a**) oleic acid, (**b**) linoleic acid, (**c**) stearic acid.

The crosslinking density of the PU film network can be quantitatively assessed by an immersion test of the crosslinked samples in toluene, and used to accurately determine the swelling degree as well as the sol fraction. As shown in Table 3, both the swelling and sol fraction of the JPU tended to decrease with OH number, indicating an increasing crosslinking density. It was found that JPU 217 exhibits the lowest swelling at 128% and the lowest soluble fraction of 25 wt.%. This sol value was approximately half of the sol fraction found in JPU 161. The presence of sol fractions indicates that some chains were not involved in crosslinking, and became more significant in JPU derived from a low OH number polyol. In JPU structures, the crosslinks are located at the middle of the fatty acid chain, e.g., at C_9_ or C_10_ and C_12_ or C_13_ for linoleic acid, and C_9_ or C_10_ for oleic acid as illustrated in Figure 6. The half pedant chain and fully saturated fatty acid chain are dangling chains in PU structures.

The crosslinking density, υ_e_ of the JPU network and the average molecular weight of the chain between cross-links, M_c_, is provided in Table 4. The crosslinking density, υ_e_, of the JPU films is in the range of 0.4–0.6, while the M_c_ is 1799 to 2593 g mol^−1^. These caused the samples to swell in toluene up to 128–231%. The M_c_ data is comparable with vegetable oil-based PU produced from a mixed polyether and polyester polyol, as reported by [38]. The samples showed an increasing crosslinking density with increasing OH number due to increased functionality of the starting polyol, ƒ. This trend is in good agreement with the reduction of M_c_ with increasing functionality reported by Zlatanic et al. [29] for PU produced from other vegetable oils such as corn, canola, linseed and soybean oil synthesised using 4,4-diphenylmethane diisocyanate (MDI). However, a lower swelling degree in the range of 40.40–96.49% reported due to relatively high υ_e_ of 1.4–1.7 and lower M_c_ of 616–776 g mol^−1^ for other oils [29].

### 3.4. Water Contact Angle

Water contact angle analysis performed to evaluate the hydrophobicity of the of the surface of the films. A high value of contact angle indicates a good hydrophobic nature of the coating, while a low value indicates that the water wets the surface. PU coatings derived from vegetable oils such canola oil, sunflower oil, and camelina oil shows a low wettability by water due to hydrophobic nature of the vegetable oil [39]. In this work, only JPU217 film exhibit a non-wetting surface character as the water contact angle is higher than 90 when exposed to water up to 10 min, as shown in Table 5. This is important aspect for coating, as water can reduce the mechanical properties of the films. Other JPU samples produced from a lower OH number polyols show hydrophilic surface character even though the ionic content is lower due to weak interactions between soft and hard segment component in JPU.

### 3.5. Water Uptake

**Figure 7:**
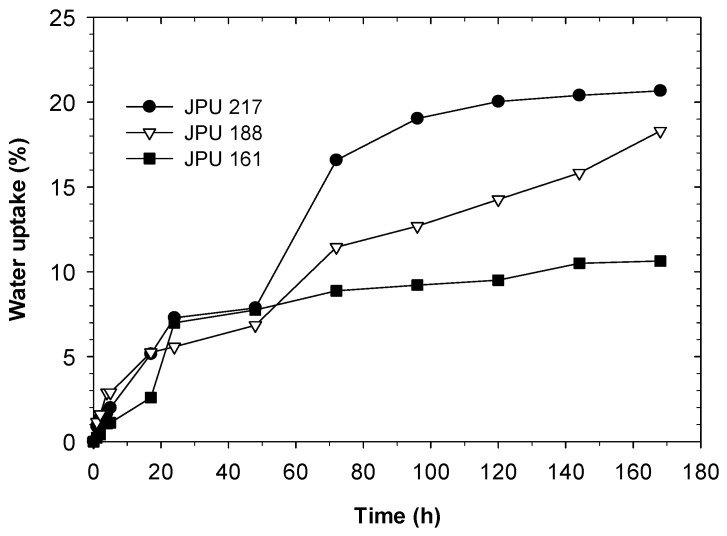
Water uptake of JPU films.

The water uptake measurements of the dried films can be used to observe the water resistance, hydrophilicity and crosslink density of crosslinked polymers formed after evaporation of water. Figure 7 shows the water uptake as a function of immersion time of the JPU films from JOLs with different OH numbers. At the early stage of immersion, the JPU films absorb water rapidly and the process slows down after 72 h. The water uptake of the samples almost reached their equilibrium at approximately 168 h, except for JPU 188. The water absorption is mainly attributed to the hydrophilic nature of the DMPA and the presence of free volume JPU films [40,41]. For comparison, the water uptake of the films at 168 h are summarised in Table 5 and correlated with hard segment and DMPA content of the JPU films. Increasing JOL OH number from 161 to 217 mgKOH/g, results in increasing water uptake from 10.6 to 20.7% in the JPU films. This was directly related to the increasing hydrophilicity due to increasing DMPA content from 4.6 to 5.4 wt.% (see Table 1), as well as the reduction of the hydrophobic JOL content from 62 to 55 wt.% in the JPU formulations.

It is also important to note that the water contact angle analysis indicated an opposite trend. Film JPU 217 has the highest hydrophobic surface characteristic and thus exhibited less wettability by water. In contrast, the water uptake of JPU 217 was the highest in this case. It is believed that immersing the films for a longer period allowed the hydrophilic groups in the film to attract the water molecules which were polar in nature [41], consequently increasing the water uptake. However, the crosslinking in all JPU films enabled the swelling behaviour to outweigh the weight loss caused by the water-soluble component in the polymer.

### 3.6. Thermal Properties by DSC

**Figure 8:**
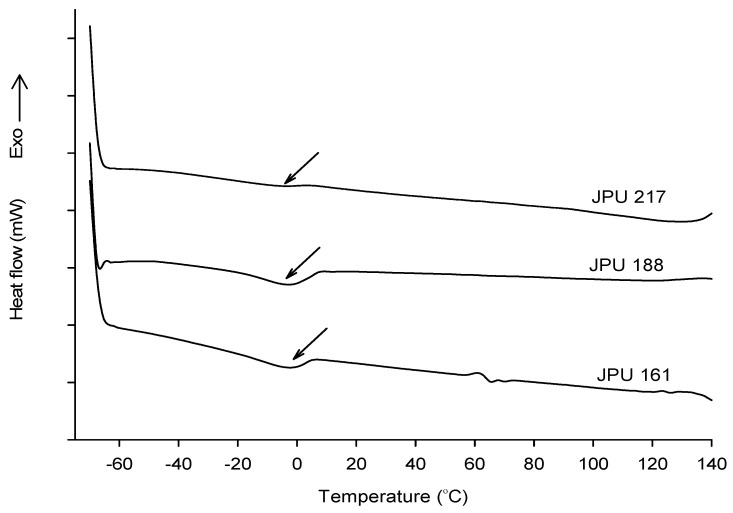
DSC thermograms of JPU films.

The differential scanning calorimetry (DSC) thermograms of the JPU films are presented in Figure 8. In DSC experiment, the glass transition temperature (T_g_) is manifested by a sudden shifted of the baseline. However, the T_g_ was not clearly observed and there is an endotherm at temperature range −10 to 5 °C shown in JPU 161 and JPU 188 indicating the samples may contain a non-functional vegetable oil which does not take part in the PU synthesis. For comparison, the T_g_ of the PUs derived from peanut, corn and soybean oil-based polyols was reported in the range of −26.6 to 3.4 °C [9].

### 3.7. Thermo-Mechanical Properties by DMA

**Figure 9:**
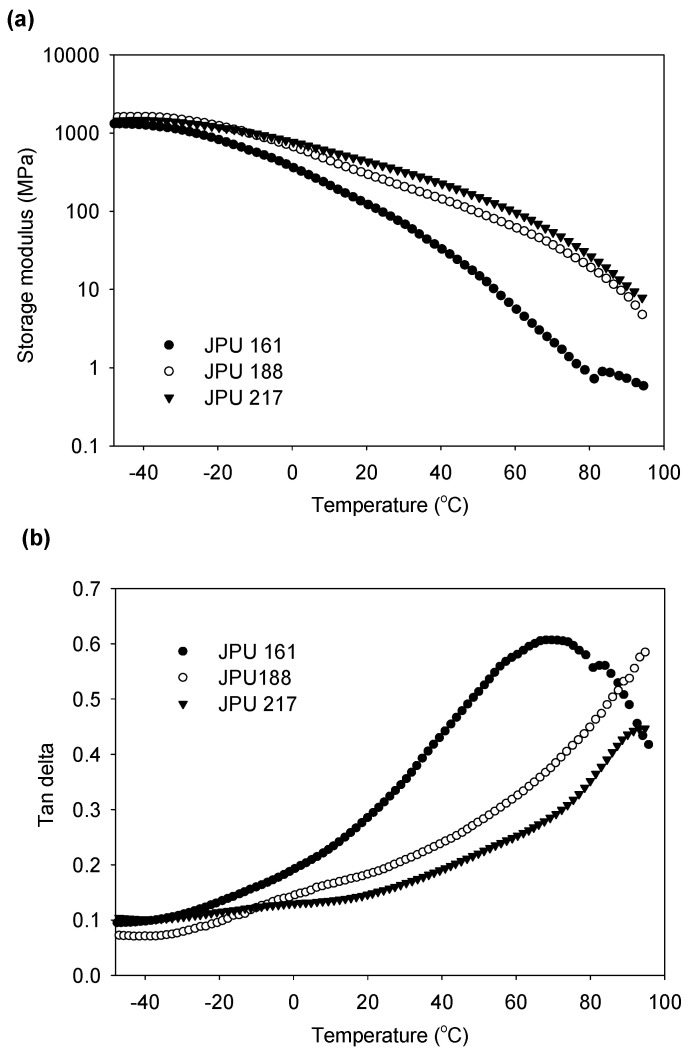
Storage modulus (**a**) and, tan delta (**b**), as a function of temperature for JPU films produced from various OH number polyol.

The dynamic mechanical behaviour of the JPU films has been investigated by use of a dynamic mechanical analyser (DMA). As compared to DSC, DMA is known to be more sensitive to the mobility of the soft segments through relaxation at the molecular level [33]. Figure 9 depicts the temperature dependence of the storage modulus and the loss factor (tan delta) of the JPU films with different hard segment content. The DMA data is summarised in Table 6. The films are in a glassy state at a temperature below −50 °C.

The magnitude of the storage modulus in the glassy state is determined primarily by the intermolecular forces and not by the strength of the covalent bonds of the polymer chain [42]. With increasing hard segment content (increasing OH numbers), the storage modulus increases due to strong hydrogen bonding in the segmental JPU. Along with the temperature rise, a drop in the storage modulus was observed. At room temperature, the storage modulus for JPU 161 was 92.8 MPa which was four times lower than JPU 217 (Table 6). The trend was consistent with the modulus of elasticity data obtained by tensile test analysis reported in our previous article [25].

The drop of the modulus corresponding to energy dissipation is shown in the tan delta versus temperature curve (Figure 9b). Many works have reported that the tan delta peak (damping peak) was associated with the glass transition temperature (T_g_). The reported T_g_ was typically higher than that measured by DSC due to the different nature of the measurement. DSC measures the heat capacity change from frozen to unfrozen chains, while DMA measures the change in mechanical response of polymer chains to heating [43,44,45]. From other perspective, Lawrence and Nelson [41] stated that the temperature of maximum damping was not a T_g_. Although it is close to T_g_, the temperature for the tan delta peak is much more sensitive to cross-link density, filler content or blend morphology rather than the T_g_ itself [42]. Since the value of Tg of the JPUs were not successfully determined by DSC analysis, the comparison could not be made. Therefore, the T_g_ for JPU films were determined from the onset of the storage modulus (Table 6).

As shown in Figure 9b, the tan delta peak for JPU 161 was observed at 69 °C. The storage modulus of JPU 188 and JPU 217 continued to drop after 95 °C, and the tan delta value increased over the entire temperature range of results, broadening the tan delta peak. The decreased height of these damping peaks with increasing OH number of starting polyol indicated an enhanced crosslinking arising from strong covalent bond interaction between the high functionality polyol and the hard segment component. High cross-linked network structure can retard and restrict the chains mobility and subsequently lead to a reduction in the damping peak intensity [41]. On the other hand, broadening of the glass to rubber transition has been reported to be associated with heterogeneity in the molecular weight between the cross-linked structure [42].

## 4. Conclusions

In this work, the bio-based polyols with OH numbers ranging from 161 mgKOH/g to 217 mgKOH/g were polymerized with IPDI, DMPA and HEMA to produce jatropha oil-based waterborne polyurethane (JPU) dispersion. The chemical and thermo-mechanical properties of the jatropha oil-based waterborne polyurethane (JPU) films were characterized. FTIR analysis suggested an increasing crosslinking in the PU films with increasing OH number polyol as a result of intermolecular hydrogen bonding interaction between hard and soft segments. Furthermore, crosslinking density measurement of the PU samples shows that both swelling and sol fraction tended to decrease with OH number. Higher crosslinking density also contributes to the hydrophobic surface character of the films, and a higher storage modulus and glass transition temperature. The JPU films are in a glassy state at any temperature below −50 °C.

## Figures and Tables

**Table 1:** Formulation of jatropha oil-based polyurethanes (JPU) dispersion.

Sample Designation	Molar Ratio				TEA (g)	Hard Segment * (wt.%)	DMPA (wt.%)
	Polyol	DMPA	IPDI	HEMA			
JPU 138	1	0.38	0.2	1.25	3.4	34	4.1
JPU 161	1	0.38	0.2	1.25	4.0	38	4.6
JPU 181	1	0.38	0.2	1.25	4.4	41	4.9
JPU 217	1	0.38	0.2	1.25	5.3	45	5.4

* Hard segment content of JPU (mass of (DMPA + IPDI + HEMA + TEA)/mass of (Polyol + DMPA + IPDI + HEMA + TEA)).

**Table 2:** Characteristic IR band of JPU film.

Wavenumber (cm^−1^)	Group	Mode
3315–3340	*st* N-H	N-H∙∙∙∙∙∙N-H
3260–3290	*st* N-H	N-H ∙∙∙∙∙∙O (ether)
2926, 2857	*st* C-H	
1730–1740	*st* C=O (urethane)	Free
1713	*st* C=O (urethane)	C=O∙∙∙∙∙∙H-N
1535	*st* C-N + N-H	
1462	δ CH_3_ or δ CH_3_	
1238	*st* N-CO-O + *st* C-O-C	
1152	*st* C-O-C	
st: stretching, δ: bending		

**Table 3:** Swelling and sol fraction of JPU films in toluene.

Sample	Density (g/cm^3^)	Swell (%)	Sol (%)	Gel (%)
JPU 161	1.041 ± 0.0006	231.9 ± 7.6	48.8 ± 5.1	51.2 ± 5.1
JPU 188	1.054 ± 0.0058	167.8 ± 17.9	44.6 ± 8.3	55.4 ± 8.3
JPU 217	1.060 ± 0.0040	128.2 ± 14.9	25.8 ± 1.1	74.2 ± 1.1

**Table 4:** Crosslinking density, molecular weight between crosslinks and solubility characteristics of the JPU films in toluene.

Sample	Hard Segment (wt.%)	Solubility Parameter, δ_2_ (J^1/2^/cm^3/2^)	Polymer-Solvent Interaction, $\chi_{12}$	Functionality, ƒ	M_c_ (g mol^−^^1^)	υ_e_, 10^3^ (mol cm^−3^)
JPU 161	38	20.09	0.31	4.51	2593	0.40
JPU 188	41	20.18	0.32	4.82	2206	0.48
JPU 217	45	20.41	0.40	8.24	1799	0.60

**Table 5:** Water contact angle and water uptake of the JPU films.

Sample	DMPA Content (wt.%)	Water Contact Angle, 1 min (°)	Water Contact Angle, 10 min (°)	Water Uptake, 168 h (%)
JPU 161	4.6	65 ± 1	56 ± 2	10.6 ± 0.7
JPU 188	4.9	66 ± 1	58 ± 2	18.3 ± 1.2
JPU 217	5.4	97 ± 3	92 ± 4	20.7± 0.3

**Table 6:** Glass transition temperature and dynamic mechanical properties of JPU films.

Sample	Hard Segment (wt.%)	T_g_ * (°C)	E’ at 25 °C (MPa)	Tan δ Peak (°C)
JPU 161	38	−34.7	92.8	69.7
JPU 188	41	−29.35	247.7	95.0
JPU 217	45	−28.61	373.8	94.6

* Based on the onset storage modulus (E’) from DMA analysis.
